# Exploring the Nutritional Impact of Sourdough Fermentation: Its Mechanisms and Functional Potential

**DOI:** 10.3390/foods13111732

**Published:** 2024-05-31

**Authors:** Zuhal Alkay, Fereshteh Falah, Hasan Cankurt, Enes Dertli

**Affiliations:** 1 Food Engineering Department, Faculty of Engineering, Necmettin Erbakan University, Konya 42010, Türkiye; zuhalalkay21@hotmail.com; 2 Department of Food Science and Technology, Faculty of Agriculture, Ferdowsi University of Mashhad, Mashhad 9177948974, Iran; fereshtefalah11@gmail.com; 3 Food Technology Department, Safiye Cikrikcioglu Vocational School, Kayseri University, Kayseri 38000, Türkiye; hcankurt@kayseri.edu.tr; 4 Department of Food Engineering, Faculty of Chemical and Metallurgical Engineering, Yildiz Technical University, Davutpasa Campüs, Istanbul 34210, Türkiye

**Keywords:** sourdough, microflora, nutritional role

## Abstract

Sourdough fermentation is one of the oldest traditional methods in food technology and occurs as a result of fermentation of flour prepared from grains. The nutritional role of sourdough is related to the final composition of fermented foods prepared through sourdough fermentation, and recently, sourdough has become an important application to improve nutrition characteristics of bread. Thanks to lactic acid bacteria (LAB) presented in sourdough microflora and metabolites partially produced by yeasts, technological and important nutritional features of the bread improve and an increase in shelf life is achieved. In addition, sourdough bread has a low glycemic index value, high protein digestibility, high mineral and antioxidant content, and improved dietary fiber composition, making it more attractive for human nutrition compared to regular bread. When the sourdough process is applied, the chemical and physical properties of fibers vary according to the degree of fermentation, revealing the physiological importance of dietary fiber and its importance to humans’ large intestine microbiota. Therefore, taking these approach frameworks into consideration, this review highlights the benefits of sourdough fermentation in increasing nutrient availability and contributing positively to support human health.

## 1. Introduction

Sourdough fermentation is a traditional method employed to enhance the nutritional, functional, and workability characteristics of cereals [1]. Utilizing sourdough for leavening is an age-old technique in grain fermentation. The process involves grinding cereals, pseudocereals, or legumes and adding water to create dough, which then transforms into sourdough over time [2]. The sourdough transformation process used for centuries is not only important for food technology but also represents an important evolution for mankind. Sourdough bread and similar products crafted from grains serve as embodiments of diverse knowledge, encompassing agricultural techniques, technological advancements, and cultural heritage. Bread holds a significant place in human sustenance, particularly in moderate climates, intertwining with tradition, societal practices, and religious beliefs. Various languages feature expressions highlighting the deep connection between life and bread, like “to earn one’s bread” or “remove bread from one’s mouth”, reflecting the enduring significance of this staple food. The etymology of words further underscores this bond, as seen in the origin of “companio” from Latin and French roots, denoting someone with whom bread is shared. Notably, in 2020, “companio” was used to name a genus of lactobacilli, *Companilactobacillus*, commonly found in sourdough cultures [3].

Sourdough, a traditional natural starter, has been utilized for centuries in the production of fermented baked goods as a substitute for baker’s yeast and chemical leavening agents. Over nearly three decades, research has gathered evidence demonstrating its effectiveness [4]. Recent advancements have seen a resurgence in the use of sourdough as the primary method for producing bread. The fermentation process of sourdough enables the creation of bread using just two ingredients—flour and water. This simplicity is highly appealing in markets where customers prefer “clean label” products [3].

**Figure 1:**
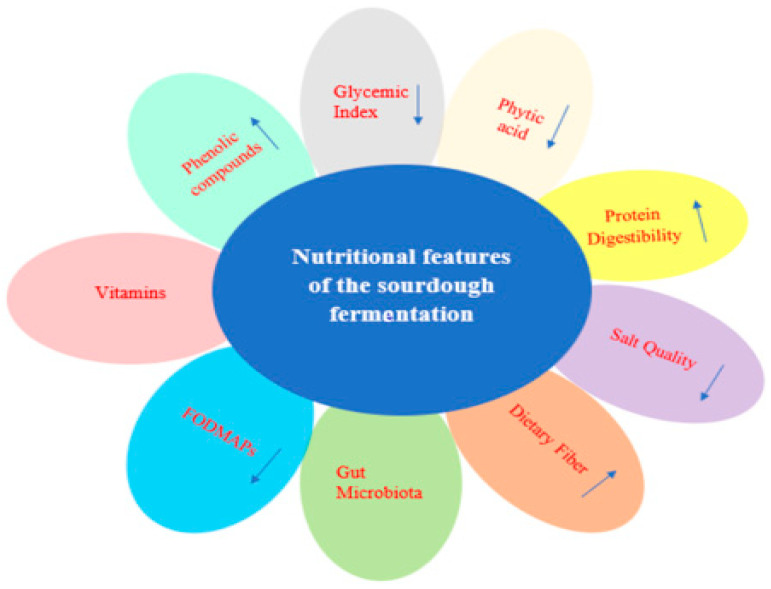
The effect of sourdough fermentation on the nutritional properties of sourdough bread. The direction of each arrow demonstrates the alteration in each component or characteristics depending on the sourdough fermentation system.

Nutritional properties of food products are typically associated with their final chemical composition and their bioactive constituents [5]. The presence of lactic acid bacteria (LAB) in sourdough contributes to the increased nutritional value of bread [6]. Sourdough not only alters the flavor of bread but also enhances its texture and provides beneficial effects on health [7]. As a result, sourdough fermentation is recognized as one of the earliest food biotechnologies utilized to ferment grain matrices, primarily valued for its influence on the sensory, structural, and shelf-life attributes of leavened bakery products [8]. A diagrammatic representation illustrating the nutritional properties of sourdough fermentation is depicted in Figure 1, which summarizes the alterations in the main dough and bread characteristics following the sourdough fermentation process. For instance, a decrement in the glycemic index of sourdough bread can be observed which is mainly associated with alterations in the starch characteristics. Similarly, sourdough fermentation results in a decrement in the phytic acid content of dough and bread that increases mineral bioavailability. Sourdough fermentation also leads to an increment in the phenolic compounds, protein digestibility, and dietary fiber content of the final bread, all of which are important nutritional properties discussed in detail below. Importantly, the positive effects of sourdough fermentation on the reduction in fermentable oligosaccharides, disaccharides, monosaccharides, and polyols (FODMAPs) as well as the salt content of sourdough have recently gained special interest, especially from the consumer’s side.

The presence of well-adapted LAB initiates the lactic acid fermentation process of sourdough, which eventually matures into a stable culture over an extended period. This culture can be obtained through three established methods, leading to the categorization of sourdough into types I, II, and III. Type I sourdough represents the traditional form that requires continuous propagation through regular refreshing with fresh flour and water. Type II sourdough involves the industrial inoculation of adapted cultures as dough acidifiers, while type III sourdough is typically dried for convenient storage and use. Type I sourdough can further be classified into subtypes Ia, Ib, and Ic based on different origins and fermentation methods. Additionally, there is a type 0 sourdough, which includes pre-doughs or sponge doughs with the addition of baker’s yeast (*Saccharomyces cerevisiae*). Manufacturers strive to identify and develop types II and III sourdough to ensure optimized and consistent product manufacturing [2]. The type of sourdough together with other process parameters can play crucial roles in determining the final nutritional characteristics of sourdough bread, and in this review, the effect of sourdough technology on the nutritional characteristics of sourdough bread will be discussed in a broad context.

## 2. Nutritional Functionality of Sourdough

### 2.1. Mineral Bioavailability and Phytic Acid

It is widely recognized that cereals, pseudo cereals, and legumes serve as significant sources of minerals such as potassium, phosphate, magnesium, and zinc. However, the utilization of these minerals is hindered by the presence of phytic acid [9]. Phytic acid possesses the ability to bind to minerals such as calcium, manganese, potassium, iron, zinc, magnesium, selenium, and copper in the digestive system, preventing their dissolution and absorption, which results in its classification as an antinutrient [10]. Phytic acid, also known as myo-inositol hexaphosphate, is present in cereals, seeds, nuts, legumes, and beans. Serving as the storage form of phosphorus in many plants (rich in six phosphate groups), phytic acid is found in varying concentrations in grains [9,10,11]. It is predominantly located in the bran portion of grains (within the aleurone layers of cereals) and exhibits strong chelating properties [10,12]. It is estimated that phytic acid constitutes approximately 1–5% of the dry mass of cereals [10].

Phytic acid functions as a proton donor, leading to the acquisition of a negative charge by phosphate groups due to the release of hydrogen ions [13]. This transformation results in the conversion of phytic acid to phytate, which contains six negatively charged phosphate groups [13]. Phytates are predominantly found in the outer layers of cereals and can be hydrolyzed by the phytase enzyme present within them [14]. Due to their strong negative charge over a wide pH range, the presence of phytates in the diet negatively impacts the bioavailability of divalent and trivalent mineral ions such as Zn^2+^, Mg^2+^, Ca^2+^, Mn^2+^, Cu^2+^, Fe^2+^, and Fe^3+^ [11]. Several enzymes are responsible for the hydrolysis of phytic acid, including soluble inorganic PO_4_^3−^, low-molecular-weight inositol phosphate, and myoinositol-releasing phytases [15]. Furthermore, phytic acid can hinder the activity of enzymes like trypsin, pepsin, α-amylase, and β-galactosidase [16]. Phytases, enzymes that degrade phytic acid, can cleave the phosphomonoester bonds of phytic acid and convert it into lower inositol phosphate esters and inorganic phosphate [1]. Only myo-inositol hexakisphosphate (IP6) and inositol pentaphosphate (IP5) negatively affect mineral bioavailability [17]. Phytase, an enzyme naturally present in grains, seeds, and microbes, including LAB, is responsible for phytic acid breakdown [1]. Aside from LAB, phytase activity is also observed in certain parts of the intestinal mucosa and in bacteria residing in the gastrointestinal tract. Phytates evade digestion as ingested phytate remains largely intact during its passage through the gastrointestinal tract, thereby impairing mineral bioavailability [10]. Additionally, the phytate–mineral complex undergoes partial hydrolysis in the human intestine due to the absence of phytate-degrading enzymes in the small intestine and the limited microbial population in the upper digestive tract [18]. Considering these factors, excessive consumption of phytic acid is believed to cause gastrointestinal issues and lead to the binding of essential minerals in food [19].

Numerous studies have demonstrated that LAB generally exhibit low extracellular phytase activity [20,21]. However, only a few studies have identified LAB strains with notable extracellular phytase activity [16,22]. Intracellular phytase enzymes are unable to access phytic acid unless the cell is disrupted. Therefore, using LAB strains with extracellular phytase activity as a starter culture in bread production has been reported to be more effective than those with intracellular phytase activity [1]. Phytase activity is influenced by various factors, including temperature, pH, proteolytic stability, and substrate specificity [16]. Recent research has suggested that the addition of sourdough yeast can enhance phytate hydrolysis [1]. It has been proposed that the significant improvement in mineral bioavailability is attributed to acidification by sourdough yeast, which indirectly stimulates the inherent phytases in the flour and enhances microbial enzyme efficacy [15].

Fekri et al. [23] reported in their study that *Kluyveromyces marxianus*, *Kluyveromyces lactis*, and *Kluyveromyces aestuarri* as yeasts, as well as *Enterococcus faecium*, *Pedostiocococus*, and *Leuconostoc citreum* as LAB, found in the sourdough yeast microbiota exhibit phytase activity and are resistant to low pH and bile effects. They observed that *K. marxianus* strains showed higher phytase efficiency compared to *S. cerevisiae* strains but had lower phytic acid content than whole wheat flour [23]. Another study conducted by Yıldırım and Arıcı [24] identified *Levilactobacillus brevis* HEB33 and *Lactiplantibacillus plantarum* ELB78 strains among the LAB isolates from sourdough samples with the highest phytase activity [24]. Furthermore, studies have shown that combining LAB and yeast strains can lead to a more than 40% reduction in phytic acid content [25]. Researchers attribute this reduction to the synergistic action of LAB and exogenous phytases produced by the yeast in the sourdough microbiota [21,26,27]. It was also noted that the endogenous phytase activity in wheat flour and sourdough mixture influences the phytase activity of microorganisms in sourdough [12,28]. The activity of endogenous cereal phytase is typically enhanced under acidic conditions during sourdough fermentation. The optimal pH range for achieving over 70% phytic acid degradation is usually between 4.3 and 4.6 [9]. In the acidic environment created during sourdough fermentation, phytase activity is usually rapid. While the optimal pH for wheat phytase is 5.0, yeast phytase functions optimally at pH 3.5 [29]. Although phytase enzymes are present in rye and wheat, they are often insufficient to significantly improve mineral bioavailability in whole-grain breads. The transformation of flour into bread leads to a decrease in phytate content due to flour phytase activity, but this reduction may not be substantial enough to significantly enhance mineral bioavailability. The decrease in phytate content during bread production is attributed to phytase activity, which is influenced by various factors such as the flour extraction rate, fermentation duration and temperature, dough acidity, and the addition of yeast and enzymes to the dough [30]. Phytate can bind directly to starch through H+ bonding with a PO_4_^3−^ group or indirectly through proteins, resulting in reduced starch solubility and digestibility [31,32]. Using sourdough technology can directly or indirectly lead to the reduction in phytate content during bread production and enhance mineral bioavailability.

### 2.2. Effect on Starch Digestibility and Glycemic Index of Sourdough Fermentation

Another benefit of incorporating sourdough is its ability to slow down the digestibility of starch in wheat flour-based products like bread [33]. This slowing down of starch digestibility is a significant nutritional concern, particularly for populations that rely on commercially leavened wheat bread in their diets. When starch content is rapidly digested and absorbed, it can lead to hyperglycemia in individuals with diabetes [34,35]. However, by utilizing sourdough, the organic acids generated during the fermentation process (such as acetic acid, which reduces gastric emptying, and lactic acid, which hinders the availability of starch through interactions with gluten) can lower the postprandial glycemic response in human blood and delay starch digestion [36,37,38]. Following sourdough fermentation, starch digestibility likely becomes a fundamental aspect in starch, grains, and cereals [39].

The macro and microstructure of grain nutrition has a significant effect on the digestibility of starch. In particular, the properties of starch are important for the glucose response [12]. Starch retrogradation, which is the recombination of amylose and amylopectin to create dual helices and likely crystal structures, favors the slow digestibility of starch. Moreover, the high branch intensity of amylopectin decelerates starch digestibility [8]. Starch digestibility may be influenced by many agents such as α-amylase binding to the substrate, gastric unloading, enzyme inhibitors, physiological parts, and the features and viscosities of digestive enzymes [40]. Amylose-rich starches have been reported to be more durable than amylolysis waxy or typical starches. It is noted that inherent starches are hydrolyzed heavily and to a restricted degree by amylases [41]. With the occurrence of gelatinization during processing, the in vitro proportion of amylolysis rises significantly [42], because the more starch gelatinizes, the faster it is digested [43]. For example, high gelatinization of starch is noted in many prevalent starchy foods like normal white wheat bread, emphasizing that the product structure is very porous. These properties cause starch to be broken down rapidly in the small intestine. In addition, it makes the blood sugar level rise very quickly [12]. However, at the product level, the glycemic response is affected by the texture integrity, porosity, and structure of starch [12], because starch digestion and the glycemic index of foods are directly related [44].

**Figure 2:**
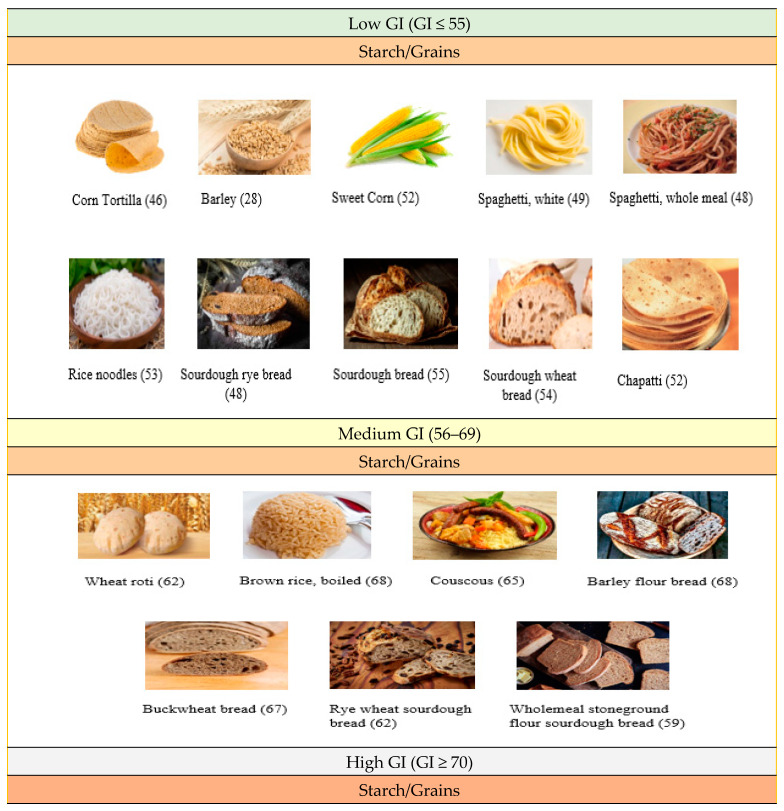

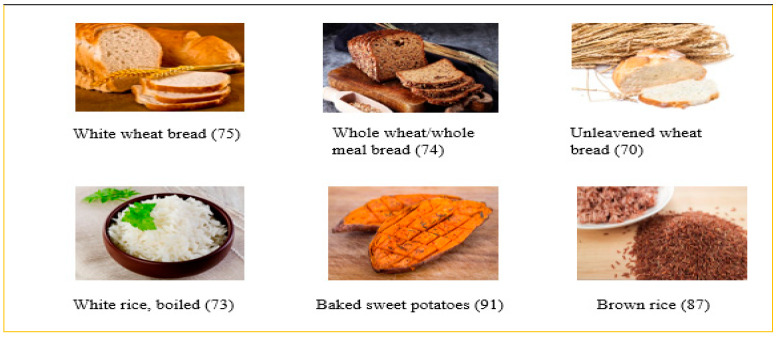
Glycemic index (GI) classification and GI values of grain-based products.

The glycemic index (GI) is defined as a method that classifies foods based on their carbohydrate content and their effect on blood sugar levels. The classification and GI values of grain-based products are shown in Figure 2 [45,46]. The GI concept is considered clinically inadequate. The glycemic index varies between foods, and these differences are not fully accounted for when consumed as part of a complex meal [47]. This is because when a complex meal is composed of a few carbohydrate sources, the impact of the lower GI component may be overshadowed by the quantity of carbohydrates from other sources. Accurate calculation of the GI of complex meals is crucial for this purpose [48]. The mechanism of action of the GI is as follows [49]:

✓ Its metabolic effects are generally related to the ratio of glucose that is absorbed from the small bowel.
✓ The ratio of glucose decreases after consumption of low-GI carbohydrate foods. For example, intestinal hormones such as incretins and insulin contribute to the reduced rate of absorption of increased postprandial glucose. Prolonged absorption of carbohydrates over time maintains the repression of free fatty acids (FFAs) and counter-regulatory reactions, resulting in lower blood glucose concentrations.
✓ A decrease in FFA concentrations over time and an increase in tissue insulinization and respiratory coefficients lead to faster withdrawal of glucose from the circulation. As a result, glucose absorption from the small intestine continues, but blood glucose concentrations return to baseline. Thus, the increase in postprandial blood glucose decreases with increasing blood glucose area above baseline [49].

The GI can be influenced not only by the raw ingredients but also by the baking methods [37]. It is suggested that products with a low or medium GI (70) are considered beneficial for health, particularly in preventing cardiovascular diseases, obesity, and type 1 or 2 diabetes [50]. Therefore, understanding the techniques behind high-GI products like bread is of primary importance [37]. One reason for which white wheat breads have a relatively high GI is that the starch becomes highly gelatinized during baking at a temperature of 250 °C. This makes it more accessible to salivary and pancreatic α-amylases [37]. It has been noted that the glycemic response of bread, classified as a high-GI product, can only be reduced through fermentation. It is observed that if the pH values of the dough are between 3.5 and 4.0, the formation of resistant starch decreases starch digestibility and, consequently, blood sugar levels [51,52,53].

Resistant starch is characterized as the sum of products that are not absorbed in the small intestine of healthy individuals, are resistant to enzymatic breakdown, and possess an exceptionally regular molecular structure [54,55]. The presence of organic acids during baking leads to the separation of branches in the amylopectin components, facilitating the formation of resistant starch [56]. This newly formed resistant starch can limit the extent of gelatinization by encapsulating the starch granules and also provides a physical barrier against the enzymatic action of α-amylases [57].

It is highlighted that the consumption of wholemeal bread, in comparison to white flour breads, leads to a reduction in GI. This reduction can be further enhanced by using sourdough [14,58,59]. Sourdough fermentation is particularly effective within low pH ranges (3.5–4.0) and contributes to decreased soluble fiber and GI [37]. Consuming sourdough bread results in lower postprandial blood sugar and insulin responses [40]. Furthermore, the glycemic response to bread can vary among individuals [60].

In both in vitro and in vivo studies, it has been shown that sourdough fermentation has the potential to reduce the GI of bread from high to medium. When sourdough fermentation is combined with the addition of dietary fiber (5–10%), the GI can drop below 55 [61]. Research indicates that sourdough fermentation using starter cultures like *Levl. brevis*, *Lpb. plantarum*, and baker’s yeast results in a bread with a higher resistant starch content compared to spontaneous sourdough fermentation. The impact of fermentation type on the estimated GI is particularly noticeable in wheat-grain bread [40]. The process of gelatinization and puffing of starch particles during sourdough fermentation is believed to increase the GI, making starch more digestible [62,63]. On the other hand, it is suggested that starch hydrolysis is hindered in the sourdough fermentation process of rye products, potentially reducing the postprandial glucose response due to the formation of the amylose layer [63]. Factors such as leavening, baking time and temperature, and flour type can also influence the starch structure and glycemic response of bread [64,65,66]. Additionally, the glycemic response is influenced by factors like starch sensitivity, protein and lipid content, and the overall structure of the food [37]. When considering the impact on the GI of sourdough breads, the type of flour used is crucial. While a decrease in GI is observed when sourdough is used in wheat breads, the use of sourdough in gluten-free breads made with buckwheat flour, quinoa, and teff may increase the estimated GI [62,65]. Researchers have noted that the use of sourdough or unleavened pseudocereal flours does not always result in a rise in heavily digestible starch. Some studies suggest that flours with smaller starch particle sizes may lead to a higher GI [61]. Various strategies have been explored to lower the GI, such as incorporating high-fiber flours like rye, oat, and barley, or adding dietary fiber supplements like β-glucans [67].

In a study conducted by Gil-Cardoso et al. [68], rats were fed different types of bread containing sourdough. These included a traditional refined wheat bread without sourdough (C-WhB), a spelt bread leavened with Rebola sourdough (Re-SpB), a durum wheat bread with Rebola sourdough (Re-DuB), and a multi-grain bread leavened with Rebola (Re-MGB), Carla (Ca-MGB), or San Francisco (SF-MGB) sourdoughs. The results indicated that rats fed with Re-SpB, Re-DuB, and Re-MGB had lower postprandial blood glucose levels compared to the control group. Additionally, rats fed with SF-MGB showed a reduced postprandial blood insulin response.

### 2.3. Effect on Protein Digestibility

In addition to the impact of sourdough on the GI, the role of LAB in processing organic nitrogen has been extensively researched [69,70]. LAB from sourdough exhibit proteolytic efficiency through cell wall proteinase, intracellular peptidases, and specific membrane transporters [8]. Simultaneous acidification also activates endogenous flour proteases, leading to progressive hydrolysis of natural proteins and an increase in the concentration of peptides and essential free amino acids (FAAs) during LAB fermentation [5]. Sourdough fermentation is considered a pre-digestion process driven by the enzymatic activity of LAB. Products containing sourdough are generally associated with improved digestibility and a higher nutritional profile in the protein fraction [71]. Proteolysis is utilized to enhance the digestibility of protein-rich flours such as seeds, reducing the amount of gluten that can trigger allergic and intolerant reactions in susceptible individuals [8], while also fortifying grain-based products [40,72].

Recent empirical and scientific studies have highlighted the ability of sourdough fermentation to enhance protein digestibility. In vitro research has demonstrated that sourdough fermentation improves the digestibility of protein, measured as the percentage of total protein dissolved after enzyme hydrolysis. A study by Rizzello et al. [73] investigated the consumption response to different types of sourdough bread among 36 healthy volunteers. The study found that sourdough bread with medium acidification increased appetite and reduced feelings of fullness, while bread with a more intense acidic flavor led to a perception of quick and high satiety. Also, they compared the digestibility of bread made with *S. cerevisiae* E10 and sourdough bread. In the results, they monitored a rise of 16% in the digestibility of sourdough bread and a rise of 18.7% in the biological amount of the proteins. Although gallbladder response did not differ between the breads, it was observed that gastric emptying was faster with sourdough bread compared to commercial leavened bread. Furthermore, it was noted that levels of essential FAAs in the bloodstream remained elevated for an extended period after consuming sourdough bread [74]. The literature suggests that sourdough bread enhances protein digestibility compared to commercial leavened bread, attributed to the proteolysis occurring during fermentation. Proteolysis breaks down proteins into bioactive peptides and amino acids, facilitating their absorption by enterocytes [53].

In recent years, there has been a growing trend of adding dried fruits to sourdough to enhance the essential amino acid content. A study found that incorporating peanut dust into flour or semolina significantly increased the lysine content in sourdough products [75]. Another study reported a notable increase in the condensation of essential amino acids like valine and methionine through sourdough fermentation with the addition of dried pears and oranges [76]. Notably, there was a significant elevation in gamma-aminobutyric acid (GABA) among the essential amino acids. GABA, a nonprotein amino acid primarily derived from the decarboxylation of L-glutamic acid, is known for its roles in neurotransmission, inducing hypotension, and exhibiting diuretic and sedative effects; so, we can prove the effects of sourdough on protein digestibility [77,78].

### 2.4. Salt Reduction Feature

Numerous studies have demonstrated that fermenting essential amino acids can help reduce the need for salt supplementation without compromising food quality [52]. High dietary sodium intake, around 10 g/day, is associated with various health risks such as cardiovascular diseases, hypertension, paralysis, stomach cancer, kidney issues, and bone demineralization [10]. Therefore, controlling blood pressure by reducing sodium intake is crucial [10]. However, reducing salt can be challenging as sodium chloride (NaCl) impacts the taste and physical characteristics of baked goods. To address this, researchers have explored various technological options [79], including salt substitutes like KCl, seasonings, flavor enhancers, malt, barley, amino acids, monosodium glutamate (MSG), and yeast products. However, it has been found that none of these alternatives provide a completely effective or convenient salt replacement strategy [52]. In this regard, sourdough fermentation is utilized to mask reduced salt content or enhance bakery products with functional anti-hypertensive components. During sourdough fermentation, sweetener FAAs and amino acid derivatives are produced. The extent of salt reduction depends on the type and strain of LAB used, with sourdough exerting stabilizing effects on them. A study revealed that incorporating rye malt sour yeast leavened with glutamate-accumulating *Limosilactobacillus reuteri* strains reduced the salt content of bread from 1.5% to 1% without compromising flavor or other quality aspects [80,81].

### 2.5. Sourdough Fermentation and Dietary Fiber

Dietary fiber consists of polysaccharides or lignins that are primarily the edible parts of plants or similar carbohydrates resistant to digestion and absorption in the human small intestine, undergoing whole or partial fermentation in the gut [8,82]. As per the American Association of Cereal Chemists (AACC), DFs are plant carbohydrates that can be fermented by colon microbes but resist hydrolysis by human enzymes, categorized into soluble and insoluble types [44]. The composition of DF varies based on factors like chemical and physical properties, including physiological effects, the polymerization rate of polysaccharides, the presence of side chains, crosslinking rate, granule size, and cell wall integrity [83]. These properties can be influenced by acidification and activation of endogenous enzymes [8]. Dietary fiber is recognized as a dietary component that significantly contributes to health improvement, with varying evidence supporting this claim [84]. It aids in lowering cholesterol and glucose levels in the blood and plays a crucial role in promoting health. There is a strong association between obesity, chronic diseases, and dietary fiber intake, with impacts varying depending on the type of fiber consumed. Whole-cereal dietary fiber is a valuable source of beneficial molecules like β-glucans, fructans, resistant starch, and arabinoxylans for the host. The bioavailability of these components in whole-cereal products often depends on specific technological factors [85]. Many consumers adhere to various processing practices, such as fortifying bread with dietary fiber, to ensure high-quality products [86]. Modern diets recommend a daily intake of 25 g or less of dietary fiber, as per the World Health Organization (WHO). Consequently, there is a focus on strategies to enhance the fiber content of foods to address this deficiency. Common sources of dietary fiber include cereals like hemicellulose, resistant starch, β-glucans, arabinoxylans, and other cereal components. Additionally, bran, wheat germ, by-products from enriching bakery products through grinding, and legume flour are gaining popularity. However, supplementing these ingredients in high quantities can alter the sensory and rheological properties of bakery products. One solution to mitigate these challenges is the use of sourdough, as it has the potential to be incorporated into bakery items, overcoming the technological drawbacks of high-fiber flours [9].

When utilizing the sourdough process, the chemical and physical characteristics of the fibers may change based on the level of fermentation [85]. Moreover, the enzymatic impact can alter the ratio of insoluble fibers to soluble fibers during bread production [87]. It has been observed that in sourdough breads, fibers can undergo two types of enzymatic hydrolysis. Specifically, different grain-specific enzymes are activated when the flour is moist, including the hemicellulase enzyme responsible for breaking down hemicelluloses [88]. Additionally, LAB can secrete enzymes with glycolytic activity that target the fibers present in the dough [14].

An in vitro study conducted by Mihhalevski et al. [89] explored the enrichment of sourdough breads with dietary fiber, specifically focusing on rye sourdough bread. The study revealed an increase in both soluble and insoluble DF ratios during the processing of rye sourdough, attributed to the biochemical and microbiological processes involved in sourdough bread production. The rise in total dietary fiber content was linked to the formation of resistant starch, while the increase in soluble dietary fiber content was associated with the conversion of insoluble fibers into soluble fibers during rye flour fermentation [89].

Another study highlighted that fermenting sourdough breads with barley enhanced their capacity, color, and sensory characteristics. This fermentation process was found to elevate the levels of dietary fiber, arabinoxylans, and β-glucans [90]. Sourdough fermentation is recognized as a suitable biotechnology for producing wholemeal rye and wheat flour products rich in dietary fiber [2,39]. Sourdough contributes to enhance the flavor, texture, and shelf life of whole-grain rye breads, as the production of sourdough wholemeal rye or wheat–rye flour blends without sourdough technology can be challenging [8]. This is because sourdough plays a significant role in traditional rye bread baking by improving workability, flavor, and structure. It is important to note that wholemeal rye bread relies on fermentation for its production [12].

Specifically, the aspartic protease enzyme, one of the endogenous rye proteases, breaks down rye proteins and secalins during rye–sourdough fermentation, generating amino acids and peptides that contribute to the bread’s flavor profile [91]. Recent studies have focused on the dietary fiber ratios in various sourdough bread varieties, with their findings summarized in Table 1.

### 2.6. Sourdough Fermentation and Gut Microbiota

The human gastrointestinal system is an extremely complex tract. This system, with its physiological ways which are highly dynamic, appears to be closely coordinated with diverse microbiota [97]. The challenge lies in the composition and functionality of the bowel microbiota, with this mutual connection persisting throughout life [98]. There is now ample evidence that compounds within the bowel microbiota help to combat entero-pathogens, ensure energy and essential nutrients, improve nutrient digestibility, and maintain optimal intestinal motility. The intestinal ecosystem also releases chemicals that play a role in immune system activity and potentially cancer prevention [99]. The intestinal microbiota seems to play a role in supplementing human nutrient metabolism and significantly contributes to the maintenance of a robust and highly active immune system [12]. The intestinal microbiota is a common term for the microbes residing in the gastrointestinal system of all vertebrates. In humans, the bowel serves as the primary habitat for a person’s microbiota. The intestinal microbiota comprises a variety of bacterial and yeast species, with its diversity and composition varying at different life stages. This variation is evident in both healthy and unhealthy individuals [100]. Furthermore, the microbial composition of the intestinal microbiota varies across different parts of the gastrointestinal system. The colon harbors a dense microbial population, while the stomach and small bowel typically have fewer bacteria. Anaerobic microorganisms make up over 99% of the bacteria in the intestine. The predominant bacterial strains found in the human intestine belong to the phyla *Firmicutes*, *Bacteroidetes*, *Actinobacteria*, *Proteobacteria*, and *Verrucomicrobia* [100,101]. It is worth noting that fermented products, particularly grain-based foods, contain numerous components that reach the gastrointestinal system and are accessible to the host’s intestinal microbes [44].

**Figure 3:**
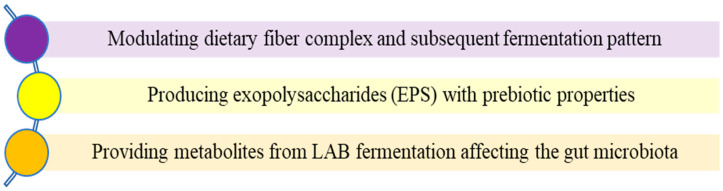
Mechanism of action of sourdough fermentation on intestinal health.

It is understood that sourdough leavening, a grain-based fermented product, along with grain fibers, increases the production of non-digestible polysaccharides in the small bowel and has the ability to slow down the digestibility of starch, which can then be fermented by the gut microbiota [102]. The impact of sourdough fermentation on bowel health operates through various mechanisms, as illustrated in Figure 3 [12,14].

Some LAB appear to produce EPSs like glucans, fructans, glucooligosaccharides, and fructooligosaccharides, which have the potential to improve intestinal health [14]. For example, levans produced by *Fruc. sanfranciscensis* have prebiotic characteristics [103]. In addition, studies have shown the formation of oligosaccharides and polysaccharides with prebiotic properties by *Liml. reuteri* LTH5448 and *Weissella cibaria* 10M in sorghum sourdough [104]. Intestinal microorganisms show the potential to metabolize these components, which have been indicated to have prebiotic features [14]. Thanks to the metabolism of these components, cholesterol and triglyceride levels decrease and insulin susceptibility is increased. In addition, propionic acid with various beneficial effects can be produced [105]. However, studies on how the consumption of sourdough bread affects not only the gut microbiota but also the leave of health-supporting metabolites along with the transition and continuation of the human gut microbiota profile are limited. In a related study, Abbondio et al. [106] fed rats a diet supplemented with sourdough breads to test the effects of sourdough on the composition and function of the bowel microbiota. It was found in this study that adding sourdough bread to the diet resulted in a reduction in low-protein diets which are desired by bowel pathogens. Moreover, some studies have indicated that the cell wall compounds of *Lpb. plantarum* found in sourdough have the capability to stimulate the immune response in the gut even when the bacteria are not viable [107]. Another significant benefit of sourdough to host health through the gut system can be the potential of the sourdough LAB as probiotics and their products as postbiotics [14]. Another study was conducted by Korem et al. [108] and in this study, volunteers were fed bread made from refined white wheat and whole wheat sourdough as dietary interventions. The effectiveness of these two bread types was compared and it was concluded that no significant differences were found between the two 1-week dietary interventions clinically, one involving the consumption of commercial white bread and the other including the consumption of sourdough bread made from whole cereals. However, person-specific factors were found to be effective for the role of bread type on clinical parameters, suggesting the role of the gut microbiota and other genetic factors as part of the response to different breads [108]. In another in vivo study, Wistar rats were fed a control diet, reconstituted whole wheat flour (white flour plus bran), commercial bread, and sourdough bread [109]. The sum cecum pond of short-chain fatty acids (SCFAs), especially the butyrate pond, has been noted to increase significantly with the consumption of unrefined products. Recent in vivo studies on the effectiveness of sourdough bread varieties on the gut microbiota are given in Table 2.

### 2.7. Sourdough Fermentation and FODMAPs

Another benefit of sourdough fermentation is its ability to reduce FODMAPs. Recent research has highlighted the importance of FODMAPs, a type of carbohydrates, in relation to overall health [14]. FODMAPs can impact a person’s health based on the amount of sugar consumed through food. When consumed in appropriate quantities, FODMAPs can have a positive effect on health (some FODMAP sugars even have a prebiotic effect) [113,114]. However, consuming FODMAP-rich foods in excess, over 20 g per day, can lead to sugar accumulation in the intestines, potentially causing various digestive issues, especially in individuals with irritable bowel syndrome (IBS) [115,116]. Since FODMAPs are not easily digestible carbohydrates, they can contribute to the development of conditions like IBS or non-celiac gluten sensitivity (NCGS) [14].

IBS is a gastrointestinal disorder that affects a significant portion of the population, with symptoms typically appearing after consuming foods high in FODMAPs. These symptoms include abdominal discomfort, stomach pain, bloating, constipation, or diarrhea [117,118]. While simple sugars and polyols have a strong osmotic effect, carbohydrates like fructans, fructooligosaccharides, and galactooligosaccharides are more prone to fermentation by the gut microbiota [116,119]. Some studies suggest that FODMAP compounds can trigger symptoms in individuals with IBS [120]. A low-FODMAP diet is often recommended by dietitians to help manage IBS symptoms [121], and the sourdough fermentation process plays a crucial role in achieving this. By utilizing the sourdough method, it is possible to reduce the levels of indigestible oligosaccharides, such as fructans and α-galactooligosaccharides, making products more suitable for individuals with NCGS and IBS [122].

During sourdough fermentation, bacterial activity can lead to the production of polysaccharides, oligosaccharides, and polyols. The breakdown of fructans during bread making is partially due to the efficiency of invertase in yeast [123]. While cereal fructans break down to some extent during regular bread making, the process is different with sourdough. The fructose produced is converted to mannitol by lactobacilli in sourdough, which is then fermented by the gut microbiota [123]. The extended fermentation period required for sourdough bread production allows for significant changes in carbohydrate composition [121].

To further reduce FODMAP content in bread, selecting specific microbes responsible for fermenting and breaking down sugars that cause gastrointestinal issues is crucial [116]. However, the extent of FODMAP reduction can vary based on the fermentation process, the type of grain used, and the amount of sour yeast incorporated into the bread dough [123]. There is limited research available on the production of grain-based products with low-FODMAP contents [44]. In a study by Struyf et al. [124], the FODMAP content of whole wheat bread was reduced by over 90% by adding *K. marxianus* and *S. cerevisiae* to the dough. It was noted that using both yeast strains together was essential to achieve a product with desirable sensory properties and a low-FODMAP content, as the control bread made with only *K. marxianus* did not produce enough carbon dioxide for the desired volume.

In their study, Laurent et al. [125] compared the impact of *K. marxianus* CBS6014 strain and *S. cerevisiae* commercial yeast on FODMAP levels. The CBS6014 strain of *K. marxianus* has been found to exhibit greater fructan hydrolysis and lower fructan content in rye sourdough bread, as well as white and whole-grain toast bread. While the presence of *K. marxianus* in sourdough rye bread led to a reduction in fructan levels, it also positively influenced the size of the bread. This highlights the importance of the *K. marxianus* strain in producing high-fiber products with a reduced FODMAP content.

In a recent study, Menezes et al. [126] examined the effects of sourdough fermentation on FODMAPs and organic acids during the production of sourdough and bread. The levels of organic acids were initially higher during the early stages of fermentation, but stabilized in the later stages. It was observed that all FODMAPs, except polyols, were significantly reduced during the process. During the initial fermentation step, sucrose was completely converted into fructose and glucose, while the concentrations of other carbohydrates decreased after the fourth step. The study highlighted that sourdough bread contained higher levels of organic acids and polyols, and lower levels of fructans, sucrose, fructose, and glucose compared to bread leavened with commercial yeast. The study suggests that sourdough fermentation could be utilized to create low-FODMAP wheat flour products, as the reduction in fructan content increased from 69% to 75%.

Schmidt and Sciurba [127] analyzed the impact of extended fermentation and the addition of sourdough on FODMAPs in final products. Their findings indicate that supplementing sourdough alters the FODMAP composition by reducing fructan content and increasing mannitol content. While breads made from refined wheat flour adhere to low-FODMAP standards, those made from rye and whole wheat flour are considered high FODMAP regardless of the fermentation process. Rye breads exceed the limits for fructans and mannitol, while whole wheat breads surpass the threshold for excessive fructose content [127].

Another study conducted by Boakye et al. [128] aimed to investigate the FODMAP contents of twenty-two different wheat cultivars grown in Minnesota, USA. The study also examined the impact of type I sour yeast fermentation (4 and 12 h fermentation periods) on FODMAP levels in these cultivars. It was found that the levels of fructans and raffinose decreased by 69% and 69%, respectively, in the sourdoughs produced with a 12 h fermentation period. However, there was a significant increase (550%) in mannitol levels observed after sourdough fermentation. These results highlight the importance of monitoring mannitol and other FODMAPs during type I sourdough fermentation, and strategies to reduce mannitol levels during fermentation and in the final product should be considered [128].

### 2.8. The Impact of Sourdough Fermentation on Vitamins

Another advantage of sourdough fermentation is its ability to enhance the vitamin content. Cereal crops contain tocopherol, vitamin B1, and folates concentrated in the seed and bran [12,39], while pseudocereals include retinol, tocopherol, and folate [129]. Certain strains of LAB are known to synthesize water-soluble vitamins from group B, such as folates B9, riboflavin B2, and vitamin B12 [14,130]. In addition to LAB, specific sourdough yeast strains have shown a significant capability to increase folate levels in rye sourdough [8]. The fermentation process with sourdough has been reported to boost folate content [131], decrease vitamin E and tocotrienol content [131], and alter vitamin B1 content depending on the processing method [132]. Therefore, the fermentation stage can impact the overall vitamin content during the baking process [132]. It is also believed that *S. cerevisiae* can promote LAB growth by producing vitamins like cobalamin, ascorbic acid, or calciferol, which are not naturally present in wheat flour [133]. While sourdough is known to enhance vitamin levels, factors such as the baking process can lead to a reduction in vitamin content [14,134]. 

### 2.9. Sourdough Fermentation and Phenolic Compounds

Phenolic compounds are secondary plant metabolites found in significant amounts in grains [135]. These components are known for their bitter flavor and their ability to hinder the digestion of starch and proteins [136]. They are synthesized through various metabolic pathways, including shikimate, phenylpropanoid, acetyl CoA, malonyl CoA, pyruvate, acetate, and amino acids like phenylalanine and tyrosine [44]. Phenolic compounds also act as antioxidants, providing various health benefits such as antidiabetic, anticancer, anti-inflammatory, antimicrobial, antioxidant, neuroprotective, cardioprotective, and hepatoprotective functions [135,137,138]. Their chemical structure allows them to scavenge free radicals and chelate metals by facilitating electron transfer or H+ donation from the OH- groups of their aromatic rings [44]. Solubility is crucial for phenolic compounds to exert their antioxidant effects in the human bloodstream [44]. Wheat and rye contain primary phenolic components like phenolic acids and alkylresorcinols, with lignans being abundant in wheat [139]. While lipophilic alkyl resorcinols in cereal bran layers do not affect bread quality, they serve as a biomarker for whole-cereal intake [140]. Ferulic acid is the main phenolic acid in rye, constituting around 50% of total phenolic acids, alongside caffeic acid, dihydrobenzoic acid, and sinapic acids [136].

Phenolic acids in wheat and rye are mostly found in connected figures and as dimers [136]. The increase in the phenolic content of cereals is provided by certain factors. These are particle size decrease, germination, supplementation of hydrolytic enzymes, and fermentation [44]. In addition, it has been found that fermentation increases the antioxidant efficiency of the phenolic part [141]. Parameters such as temperature during fermentation, final pH, time, microorganisms added, grain kind, and cereal texture play a significant part in the results related to the leave of bound phenolics [44]. Among the added microorganisms, LAB phenolic acid metabolism is achieved through reductases and decarboxylases. Subsequently, decarboxylating hydroxybenzoic and hydroxycinnamic acids to phenol or vinyl derivatives takes place. Hydroxycinnamic acids and their vinyl derivatives are transformed by reductases that hydrogenate the double bond. The bioconversions of phenolic acids are generally species-specific [136].

There are limited studies on the addition of grain enzymes and specific starter cultures to facilitate the transformation of phenolic compounds in sourdough fermentation [136]. The significance of phenolic components in sourdoughs stems from the presence of these compounds in wheat flour [139]. Both whole- and white wheat flour contain some phenolics that may not be readily bioavailable [139]. However, natural fermentation can enhance the bioavailability of these phenolics. This is because yeast microbes produce enzymes that break down insoluble phenolics, making them more accessible [139]. It has been observed that phenolic compounds in flour undergo changes during fermentation.

In previous studies, the antioxidant potential of a wheat bran–flour mixture and rye flour was compared with sourdough fermentation. It has been reported that the antioxidant efficiency following sourdough fermentation surpasses that of traditional products [142]. However, research indicates that in addition to enhancing antioxidant capacity, sourdough fermentation also hinders the performance of α-amylase and α-glucosidases, impacting their activity in starch hydrolysis [143,144].

## 3. Conclusions

In conclusion, this comprehensive evaluation of the nutritional role of sourdough fermentation has shed light on its diverse mechanisms and potential functional aspects. Through this study, the significant impact of sourdough fermentation on various nutritional factors, including mineral bioavailability and phytic acid, starch digestibility and glycemic index, protein digestibility, salt reduction, dietary fiber content, gut microbiota modulation, FODMAPs, vitamins, and phenolic compounds, has been explored. The findings highlight the multifaceted benefits of sourdough fermentation in enhancing nutrient availability, promoting digestive health, and potentially contributing to overall well-being. Further research in this area could provide valuable insights into optimizing the nutritional quality of fermented foods for improved dietary outcomes.

## Figures and Tables

**Table 1:** Studies on dietary fiber ratios in sourdough bread varieties in recent years and total dietary fiber amounts in results.

Reference	Flour Type	Microorganism	Total Dietary Fiber Ratio Determined as a Result
Saa et al. [85]	Kamut khorasan and durum wheat-grain flour (milky and entirely mature)	*Lpb. plantarum*, *Fructilactobacillus sanfranciscensis*, *Levl. Brevis,* and *S. cerevisiae*	Khorasan flour at immature phase using sour yeast fermentation at high temperatures resulted in (10.26 mg/100 g). Khorasan flour at entirely mature phase using sour yeast fermentation at high temperature resulted in (19.25 mg/100 g). Breads acquired with durum wheat flour at entirely mature phase using industrial fermentation at high temperatures resulted in (15.48 mg/100 g).
Pejcz et al. [90]	Wheat flour and a wheat–barley blen	*Saccharomyces chevalieri*, *Lacticaseibacillus casei,* and *Levl. brevis*	Barley sour yeast ended in a higher condensation of both dietary fiber and the arabinoxylans and β-glucan fractions compared to barley whole wheat. Total dietary fiber was 10%.
Olojede et al. [92]	Sorghum flour	*Pediococcus pentosaceus* SA8 and *S. cerevisiae* YC1	The highest total dietary fiber amount (17.2%) was found in sour yeast bread made with *P. pentosaceus* SA8 and *S. cerevisiae* YC1 strains.
Çetin-Babaoğlu et al. [93]	Sourdough breads are prepared from immature wheat flour (26 and 36 days)	*Liml. reuteri*, *Levl.* brevis, *Lbp. plantarum*, *Liml. Fermentum,* and *Lacticaseibacillus rhamnosus*	Immature wheat sourdough bread (26 day) resulted inm2.18%. Immature wheat sourdough bread (36 day) resulted in 2.10%
Das et al. [94]	Millet flours such as kodo, barn, small, and foxtail were used for sourdough bread production	Sourdough starter culture mix	Foxtail 20–50%, refined flour 65–35%, chickpea flour 10%, and tapioca flour 5%.
Olojede et al. [95]	Sorghum flour and corn starch were used	*P. pentosaceus* and *Weissella confusa*	The highest total dietary fiber amount (15.9%) was found in sourdough bread with *P. pentosaceus*, while the lowest total dietary fiber amount was observed in the control bread without sour yeast (13.25%).
Subaşı and Ercan [96]	Whole wheat flour (Tosunbey, Kenanbey, İkizce-96, Bezostaja-1)	*Lpb. Plantarum* and *Fruc. sanfranciscensis*	Whole wheat bread of Bezostaja-1 had the highest total dietary fiber amount (15.94%).

**Table 2:** Recent in vivo studies on the influence of sourdough bread varieties on the gut microbiota.

	Human Studies	
Reference	Type of Flour, Type of Sourdough Bread Made with LAB, and Number of Volunteers	Results for Gut Microbiota
Da Ros et al. [98]	Wheat flour, *Lpb. plantarum* CR1, *Furl. rossiae* CR5, and *S. cerevisiae* E10, 40 healthy volunteers	The amount of SCFAs and isovaleric and 2-methylbutyric acids increased.
	**Animal Studies**	
**Reference**	**Type of Sourdough Bread and Type of Animal Used**	**Results for Gut Microbiota**
Arias et al. [110]	Celta bread, *S. cerevisiae*, *S. pastorianus*, *C. sakei*, *Lpb. paralimentarius*, *P. parvulus*, *Levl. Brevis,* and *Leu. citreum*, 10 female 8-week-old C57BL/6 J mice.	In general, after bread ingestion, there was a significant reduction in the *F.* phylum relative to the baseline. There was a substantial rise in *Bacteroidetes* bacteria in the intestinal microbiota of the mice fed with the commercial bread. In the group fed with sour yeast bread in the Celta class, the main change was related to *Verrucomicrobiaphylum*.
Koistinen et al. [111]	Whole-grain wheat and whole-grain rye, *C. milleri*, *Levl. Brevis,* and *Lpb. plantarum*, C57BL/6 J male mice (*n* = 74)	It has been noted that diets enriched with bran produce a rise in the relative amount of a few bacterial taxa, like *Akkermansia*, *Bifidobacterium*, *Coriobacteriaceae*, *Lactobacillus*, *Parasutterella*, and *Ruminococcus*.
Kwon et al. [112]	Yeast-leavened white bread and sourdough bread, male C57BL/6 mice	Mice fed with sourdough bread showed a diabetes-lowering effect by decreasing the GI, owing to the existence of dietary fiber and SCFAs. Some useful bowel bacteria like *Akkermansia*, *Bifidobacterium, and Lactobacillus* were increased in mice in the sourdough bread-fed group.

## Data Availability

The original contributions presented in the study are included in the article, further inquiries can be directed to the corresponding author.
